# Pursuit of precision oncology in the treatment of metastatic hormone receptor–positive breast cancer: Making strides or barely moving?

**DOI:** 10.3322/caac.70060

**Published:** 2026-01-12

**Authors:** Ilana Schlam, Ajay Dhakal

**Affiliations:** ^1^ Dana‐Farber Cancer Institute Harvard Medical School Boston Massachusetts USA; ^2^ Wilmot Cancer Institute University of Rochester Medical Center Rochester New York USA

Breast cancer is the most common cancer among females in the United States, and metastatic breast cancer is estimated to cause more than 42,000 deaths in 2025.^1^ Hormone receptor (HR)–positive, human epidermal growth factor receptor 2 (HER2)‐negative metastatic breast cancer is the most common subtype of metastatic breast cancer.^1^ Although advances in endocrine and targeted therapies have improved patient outcomes, resistance and disease progression remain common, and the optimal sequencing of available treatments is unknown. In their review, Raghavendra and colleagues present a framework for managing patients with HR‐positive, HER2‐negative metastatic breast cancer, describing knowledge gaps and the growing role of precision oncology tools, such as genomic profiling of tumor tissue or circulating tumor DNA (ctDNA), in treatment selection.^2^ The review describes advances in endocrine and targeted therapies that have improved patient outcomes.

As highlighted in the article, there have been two major advances in the treatment of HR‐positive metastatic breast cancer over the last decade: first, the approval of cyclin‐dependent kinase 4 and 6 (CDK4/6) inhibitors and, second, the increased use of genomic profiling of tumor tissue and ctDNA for treatment selection, driven primarily by the approval of novel, molecularly targeted therapies that were previously unavailable. An improved understanding of the estrogen receptor pathway and its downstream promotion of the cell cycle has led to the development and approval of CDK4/6 inhibitors in combination with endocrine therapy.^3^, ^4^, ^5^, ^6^ The introduction of these novel drugs is associated with significant improvements in progression‐free survival and, in many patients, overall survival as well, making it one of the biggest advancements in the treatment of metastatic HR‐positive breast cancer.^4^ However, some patients do progress early on these therapies, and, as Raghavendra and colleagues have described, it is unclear whether CDK4/6 inhibitors need to be introduced in the first‐line setting for all patients.^7^ To improve precision, we need to develop biomarkers that help identify those patients who have intrinsic resistance to CDK4/6 inhibitors and those who can safely forego the addition of CDK4/6 inhibitors in the first‐line setting.

A major shift in recent years has been the routine integration of genomic and molecular characterization using next‐generation sequencing (NGS), including analyses of tumor tissue and ctDNA. These tools provide insight into acquired resistance mechanisms, guide targeted therapy selection, and recent data suggest that adaptive treatment changes based on the emergence of genomic alterations, before clinical or radiographic progression, could improve patient outcomes.^8^, ^9^

NGS has become the standard for evaluating metastatic HR‐positive/HER2‐negative disease, particularly at disease progression after CDK4/6 inhibitors or in patients whose tumor progresses during or soon after the completion of adjuvant endocrine therapy. The detection of *ESR1* mutations, acquired in approximately 40% of patients previously treated with aromatase inhibitors, has direct therapeutic implications, guiding the selection of selective oral estrogen receptor degraders, particularly elacestrant and imlunestrant.^10^, ^11^ The identification of *PIK3CA* mutations allows clinicians to offer PI3K and AKT pathway inhibitors, such as inavolisib and capivasertib.^12^, ^13^ Importantly, NGS results are dynamic because HR‐positive/HER2‐negative tumors evolve under treatment pressure, and patients often benefit from periodic molecular reassessment to identify acquired, potentially targetable mutations; however, the optimal frequency of testing is unknown. At this time, testing is indicated only when results might inform treatment options. In selected patients, even when molecular data do not directly assign a single optimal option, it refines the decision‐making framework. For instance, the absence of *ESR1* mutation after progression on aromatase inhibition raises the likelihood of continued endocrine sensitivity. Conversely, high metastatic tumor mutational burden may identify individuals who are more likely to respond to immunotherapy.^14^

The development of ctDNA analysis has increased the feasibility of repeated molecular profiling across the disease course. Liquid biopsy allows noninvasive detection of emerging resistance mutations, sometimes months before radiographic or clinical progression. This offers the potential to intervene earlier. The PADA‐1 and SERENA‐6 trials (ClinicalTrials.gov identifiers NCT03079011 and NCT04964934, respectively) suggest that switching from an aromatase inhibitor to a selective oral estrogen receptor degrader at the time of *ESR1* mutation detection in ctDNA can prolong progression‐free survival without requiring clinical or imaging evidence of progression.^8^, ^9^ Whether these translate into gains in overall survival requires further follow‐up. Even so, these studies represent a shift in thinking toward molecularly adaptive therapy, although the optimal timing and appropriate clinical end points for routine implementation in the clinic remain under active research. Studies that include crossover and carefully selected end points are needed to ensure that any observed benefit of a treatment switch driven by mutation emergence reflects an actual improvement in patient outcomes rather than an apparent effect driven by lead‐time bias.

The review by Raghavendra and colleagues underscores that progress in HR‐positive/HER2‐negative metastatic breast cancer is now focused on precision medicine. The increasing availability of NGS enables clinicians to anticipate and respond to tumor evolution in ways that were previously not possible. The challenge ahead is to refine when and how to apply these tools, interpret them in a clinical context, and ensure that treatment personalization is grounded not only in molecular signatures but also in each patient's priorities. In the pursuit of precision oncology, the management of HR‐positive metastatic breast cancer has made strides in the last decade. However, improved knowledge reveals larger or more complex gaps. Hence, despite making strides, it does appear that we need to do more than ever in the pursuit of precision oncology, in our quest to find the right treatment for the right patient with HR‐positive metastatic breast cancer.

## CONFLICT OF INTEREST STATEMENT

Ajay Dhakal reports grants/contracts from Celucity and Puma Biotechnology; personal/consulting fees from AstraZeneca, Gilead Sciences Inc., and WebMD; and support for other professional activities from MJH Life Sciences outside the submitted work.

Ilana Schlam reports personal/consulting fees from AstraZeneca and Novartis outside the submitted work.
